# Editorial: Beta Amyloid: From Physiology to Pathogenesis

**DOI:** 10.3389/fnmol.2022.876224

**Published:** 2022-03-14

**Authors:** Robert A. Nichols, Walter Gulisano, Daniela Puzzo

**Affiliations:** ^1^Department of Cell and Molecular Biology, University of Hawaii, Honolulu, HI, United States; ^2^Department of Biomedical and Biotechnological Sciences, University of Catania, Catania, Italy

**Keywords:** beta amyloid, Alzheimer's disease, pathogenesis, synaptic plasticity, cognitive deficits, neuroinflammation, neurogenesis

The neuropeptide beta amyloid (Aβ) is present in the brain throughout life, but accumulates with age, with levels rising dramatically years prior to the diagnosis of Alzheimer's disease (AD) (Bateman et al., 2012). In AD, elevated levels of Aβ assemble into extracellular plaques, notable histological hallmarks of the disease along with accumulation of intracellular neurofibrillary tangles and neuronal loss in select regions of the brain. Curiously, the level of Aβ at synapses is regulated by nerve activity and the pool of Aβ in brain turns over at a remarkable rate (Cirrito et al., 2005; Bateman et al., 2006). These observations, and others, led to the postulate that in the absence of dementia the low levels of soluble oligomeric Aβ present in brain impact synaptic and neural circuit function, now borne out in a range of functional studies (Puzzo et al., 2008, 2012; Morley et al., 2010; Lawrence et al., 2014; Gulisano et al., 2019).

The objective of this Frontiers Research Topic collection was to bring together a cross-section of reports on Aβ as a physiological regulator in relation to studies on the contribution of Aβ pathology to AD pathogenesis. A fundamental issue in the study of AD pathogenesis revolves around the role of supranormal levels of Aβ arising during the prodromic period before AD, linked to synaptic dysfunction and, ultimately, synapse loss as well as neural circuit hyperactivity and select cognitive dysfunction, which are to varying extents inter-related to neuroinflammation. Approaches to lower pathological levels of Aβ in AD would thus be expected to normalize function, but, for humans, has largely failed in the vast majority of clinical trials. This conundrum underscores the need to better understand all aspects of Aβ function and regulation, informing new approaches, possibly identifying new targets and, especially, avoiding misleading methodological and conceptual pitfalls.

For this Research Topic we received a large number of submissions from which 14 articles by a total of 70 authors from 10 countries were published. The articles included seven original research papers along with a diverse array of reviews, mini-reviews and perspectives. These articles range from confrontation of the conceptual and methodological pitfalls in AD research (Puzzo and Conti) to Aβ regulation of synaptic function (Fagiani et al.; Forest et al.; Karisetty et al.; Guan et al.), neural circuitry (Hector and Brouillette), organelle trafficking (Fabbrietti et al.), transcriptional regulation (Jesko et al.); neuroepigenic gene regulation (Karisetty et al.), neurogenesis (Li Puma et al.); glia and neuroinflammation (Guzman et al.; Spampinato et al.; Oberstein et al.; Seol et al.); and cognitive processes (Zhang et al.; Guan et al.). This wide range of topics underscores the breadth of impact of Aβ as a synaptic, neuronal, neuroimmune and cognitive regulator.

The Perspective by Puzzo and Conti confront many of the limitations, experimenter bias, conceptual bias and paucity of rigor and reproducibility in methodology. Of particular note is over-reach in regard to the experimental model under study, particularly in preclinical studies using rodent models that have repeatedly failed to translate to the clinic, wherein intrinsic variables are not properly considered. For Aβ in AD, *confirmation* bias, extrapolation from preclinical rodent models and dismissing a role for Aβ out of hand have compromised the need for open, neutral, question-driven research on the fundamental role for Aβ in the brain as a foundation for understanding Aβ in AD pathogenesis.

As the progressive elevation of Aβ levels appear to correlate with synaptic dysfunction, new insights into synaptic regulation by Aβ are reviewed by Karisetty et al. in which both direct action on the synapse and neuroepigenetic regulation of synaptic genes contribute to synaptic impairment, focusing on DNA methylation and histone acetylation. The current status of selective demethylase and HDAC inhibitors in clinical trials is also reviewed. Similarly, Fagiani et al. review the impact of Aβ on synaptic function but from the point of view of Aβ as endogenous regulator, with elevation levels leading to a disruption of synaptic homeostasis as one of the earliest stages in AD pathogenesis, separate but related to Aβ disruption of synaptic plasticity.

Delving into molecular mechanisms for synaptic dysregulation by Aβ, Guan et al. reviewed the current understanding of the role for calcium, ranging from neuroinflammatory processes with a focus on proinflammatory cytokines, neuronal apoptosis in relation to ER and mitochondrial calcium dysregulation, dysregulation of neurogenesis, excitotoxicity, lysosomal degradation pathways and autophagy. Zhang et al. demonstrated that glucocorticoids can accelerate disease development and progression in a mouse model of AD by interfering with plasticity-related proteins and apoptotic pathways, indicating a potential detrimental effect of long-term cortisone treatments. Jésko et al. found that a sphingosine-1-phosphate receptor modulator reversed, in part, synaptic dysfunction in an aged familial AD mouse model. These findings provide new insights into the disruption of sphingolipids in AD, which appears to be an early event in disease progression. Continuing with approaches with therapeutic potential, Forest et al. demonstrated that the neuroprotective N-terminal Aβ core hexapeptide, YEVHHQ, potently reversed impaired synaptic plasticity in AD pathology models in manner dependent upon PI3 kinase via mTOR, in addition to its previously noted protection against neuronal toxicity.

In a parallel with synaptic dysfunction, Aβ induces neurite atrophy and compromised neurite trafficking. Fabbrietti et al. addressed fundamental mechanisms in Aβ-triggered dendrite atrophy by studying trafficking of Golgi-like organelles, essential for dendritic arborization, tagged for live imaging. Aβ treatment led to rapid reduction in Golgi-like organelle trafficking, most pronounced in higher order dendritic arbors, and a tetracyclic antidepressant with neurotrophin-like activity was able to rescue the Aβ-induced reduction in trafficking concomitant with reversal of neuritic atrophy, offering a novel means to prevent or reverse this very early Aβ-linked neuropathology.

Moving to the level of neural circuits, Hector and Brouillette review neuronal network hyperactivity in rodent AD pathology models as well as humans with mild cognitive impairment, as an early pathological event in AD pathogenesis, replicated in *in vitro* studies applying Aβ. While a number of mechanisms may contribute to an excess of excitation in neural circuits leading to hyperactivity, several factors converge on altered glutamatergic signaling, suggesting a range of potential sites for intervention.

Examining the impact of neuroimmune responses catalyzed by direct application of Aβ by injection into the CA1 region of the hippocampus, as a highly simplified *in vivo* model, Guzman et al. found rapid mobilization of reactive astrocytes and microglia as well as endothelial dysfunction, correlated with spatial memory deficits, well before onset of tau-based pathology or neurodegeneration. Spampinato et al. addressed more broadly the cross-talk between neuroinflammation and the peripheral immune system as AD develops, particularly in the context of a compromised blood-brain barrier (BBB) in AD allowing lymphocyte infiltration. Using *in vitro* models, it was found that Aβ-primed reactive astrocytes altered CD4+ T cell cytokine and neurotrophin expression, and in turn, the CD4+ cell moderated the inflammatory response of the Aβ-primed astrocytes. The latter finding is intriguing as a possible transient mitigating impact of the peripheral T cells on the Aβ-triggered inflammatory response in the astrocytes at the BBB. Regarding the interaction of astrocytes with Aβ, specifically intracellular Aβ degradation leading to the formation of N-terminally truncated forms of the amyloid peptide, which are prominent in extracellular amyloid deposits, Oberstein et al. examined the effect of deletion or inhibition of the degradative enzyme cathepsin B and observed a sharp reduction in the amount of secreted N-terminal truncated Aβ, while secretion of full-length Aβ increased. These findings add complexity to understanding of the degradative pathways for internalized Aβ, indicating that the N-terminal truncated forms of Aβ are generated outside of the lysosomal compartment. Turning to microglial responses to Aβ, Seol et al. reviewed cell-based Aβ clearance as a limited compensatory response in contrast to a developing feed-forward response of microglia to advancing Aβ pathology, wherein induction of the inflammasome contributes to seeding and spreading of Aβ pathology via released ASC-Aβ complexes, providing another unique target for intervention in AD pathogenesis.

Lastly, Li Puma et al. reviewed the controversial association of Aβ accumulation with the development of impaired neurogenesis. As to whether organized neurogenesis continues into adulthood in humans remains in question; however, well defined AD pathology models indicate that altered hippocampal neurogenesis occurs prior to dysfunction in memory formation, and more importantly, suggests that increased hippocampal neurogenesis would improve memory acquisition.

Altogether, we hope that this collection of reviews and original research for this Research Topic provides new perspectives and insights into the wide range of connections between Aβ as regulator of multiple physiological processes in the brain and supranormal Aβ in AD pathogenesis.

## Author Contributions

All authors listed have made a substantial, direct, and intellectual contribution to the work and approved it for publication.

## Conflict of Interest

The authors declare that the research was conducted in the absence of any commercial or financial relationships that could be construed as a potential conflict of interest.

## Publisher's Note

All claims expressed in this article are solely those of the authors and do not necessarily represent those of their affiliated organizations, or those of the publisher, the editors and the reviewers. Any product that may be evaluated in this article, or claim that may be made by its manufacturer, is not guaranteed or endorsed by the publisher.
